# Thinning‐induced canopy opening exerted a specific effect on soil nematode community

**DOI:** 10.1002/ece3.3901

**Published:** 2018-03-14

**Authors:** Bing Yang, Xueyong Pang, Weikai Bao, Kexin Zhou

**Affiliations:** ^1^ Key Laboratory of Mountain Ecological Restoration and Bio‐resource Utilization of Chinese Academy of Sciences & Ecological Restoration and Biodiversity Conservation Key Laboratory of Sichuan Province Chengdu Institute of Biology Chinese Academy of Sciences Chengdu China; ^2^ Nanjing Institute of Environmental Sciences Ministry of Environmental Protection Nanjing China

**Keywords:** abundance, diversity, nematode, soil food web, thinning

## Abstract

Changes in microclimate, soil physicochemical properties, understory vegetation cover, diversity, and composition as well as soil microbial community resulting from silvicultural practices are expected to alter soil food webs. Here, we investigated whether and how contrasting‐sized canopy openings affect soil nematode community within a 30 year‐aged spruce plantation. The results indicated that the responses of soil nematodes to canopy opening size were dependant on their feeding habit. The abundance of total nematodes and that of free‐living nematodes was negatively correlated with soil bulk density, whereas the abundance of omnivore–predators was negatively correlated with soil bulk density and shrubs cover, respectively. The ratio of the sum abundance of predators and omnivores to the plant parasites’ abundance, Simpson's dominance index, Pielou's evenness index, and sigma maturity index, maturity index (MI), MI_2‐5_, basal index, enrichment index, and structure index was sensitive to alteration in canopy opening size. Multivariate analysis indicated that thinning‐induced gap size resulted in contrasting nematode assemblages. In conclusion, soil nematodes should be integrated as an indicator to monitor soil multifunctionality change due to thinning.

## 1 INTRODUCTION

Spruce plantation replaced most of the old‐growth forests in the western part of Sichuan Province during the 1970–1980s. However, this even‐aged plantation has become a prime of low yielding forest characterized by sporadic understory vegetation and thick layer of undecomposed litters. However, understory vegetation is crucial to maintain plant forest diversity (Ares, Berryman, & Puettmann, 2009) and to deliver multiple ecosystem services (Nilsson & Wardle, 2005), such as providing shelter and food to animals (Dodd et al., 2012; Taki et al., 2010), maintaining soil physicochemical property and biological activity (Fu et al., 2015; Jonathan et al., 2016). Thus, sustainable silvicultural practices that have potential in enhancing understory plant diversity are needed in plantations in this region.

Small canopy openings are ubiquitous in natural forest ecosystem (Clinton, 2003) especially boreal coniferous forest, and canopy openings usually occur in response to natural disturbance such as windstorm, ice storms, insect‐attacks, pathogens, and/or human activities, for example, stand‐replacing fires, thinning, or logging. Additionally, the structure and spatial complexity of canopy plants are expected to affect understory species abundance and richness by inducing shifts in resource availability and competitive relationships (Berger & Puettmann, 2000), and the vegetation cover and richness increased with size of the canopy openings (Trentini et al., 2017; Wang & Liu, 2011). Therefore, thinning is adopted as a common management practice to produce highly heterogeneous stands in even‐aged plantations (Arseneault & Saunders, 2012).

Group thinning affected forest landscapes temporally and spatially (Klingsporn, Webster, & Bump, 2012), and implementing different thinning intensity to plantations provides an opportunity to mimic the succession dynamics. Numerous studies revealed that gap creation in natural forests would profoundly affected microclimatic variables (Muscolo, Sidari, & Mercurio, 2007), fine root growth (Bauhus & Bartsch, 1996; Jones, Mitchell, Stevens, & Pecot, 2003), plant species composition (Brokaw & Busing, 2000; Chávez & Macdonald, 2010; Fahey & Puettmann, 2008), soil nutrient status, and soil microbial communities (Lewandowski et al., 2015; Schliemann & Bockheim, 2014; Yang, Pang, Hu, Bao, & Tian, 2017). However, available studies addressing how soil fauna would respond to shifts in these variables due to thinning are scare.

Nematodes are one group of the ubiquitously distributed soil invertebrates with extremely high density and diversity occupying multiple trophic groups in soil food webs. Additionally, free‐living nematodes play a crucial role in soil organic matter decomposition, nitrogen mineralization, and nutrient cycling (Liang et al., 2009). Thus, soil nematode community provides crucial information about soil biological activity and contribution in associated ecosystem processes (Bongers & Bongers, 1998; Tsiafouli, Bhusal, & Sgardelis, 2017; Zhao & Neher, 2013). In forests, soil nematode community is regulated by soil, plant (including plant identity, diversity, stand age as well as litter depth (De Deyn, Raaijmakers, van Ruijven, Berendse, & van der Putten, 2004; Williamson, Wardle, & Yeates, 2005; Cesarz et al., 2013; Zhang et al., 2015), and forest management (Zhang et al., 2015; Zhao et al., 2013, 2014).

As aforementioned, gap formation significantly affected microclimate, soil nutrient, plant community, and soil microbial profiling, we hypothesize that canopy openings resulting from thinning would exert significant impacts on the abundance, richness, diversity, and community structure of soil nematodes. However, to our knowledge, there have been two empirical cases (Bjørnlund & Christensen, 2005; Ritter & Bjørnlund, 2005). Bjørnlund and Christensen (2005) reported that site heterogeneity rather than litter quality accounted for different responses of nematode functional groups to gap openness in the early stage of litter decomposition. Ritter and Bjørnlund (2005) found the effects of gap formation on nematodes depended on gap age and the trophic groups of nematodes, and the likely reason lies in the difference in carbon input from living roots. Furthermore, the effects of thinning on microclimate, plant community, and soil microbial community are dependent on other environmental factors. For instance, the effects of thinning on microclimate varied with the topography and evergreen understory (Clinton, 2003). Besides, the effects of gap formation on soil microbial community showed considerable variations across ecosystems and ecotones (Lewandowski et al., 2015; Schliemann & Bockheim, 2014). Finally and foremost, the trophic level of soil organisms in the soil food webs would lead to differential effects of abiotic factors on soil organisms. For instance, soil temperature and moisture disproportionately affected bacterivorous nematodes and bacterial community (Papatheodorou, Argyropoulou, & Stamou, 2004). Therefore, the effect of gap on soil fauna is still elusive. Since soil nematode is one group of the most soil fauna occupying crucial role in soil food webs, to address the potential effects of gap size on soil nematode community with a case study is necessary.

The major objectives of this study were to explore whether and how gap size would affect soil nematode community. As links between soil nematodes and environmental factors seem to be correlated with the feeding habits of nematodes, we hypothesized that the effects of gap openings on of nematodes abundance would vary with the feeding habits of nematodes. We also hypothesized light‐medium thinning will not induce shift in soil nematode community, whereas heavy thinning would resulted a apparent compositional and functional changes for soil nematodes due to the changes in microclimate, soil biogeochemistry, plant community, and plant–soil interactions. We further hypothesized that the properties of soil nematodes were more sensitive than soil physicochemical and microbial properties in indicating the shifts in soil ecological processes resulting from gap creation.

## 2 MATERIALS AND METHODS

### 2.1 Study area

The study was conducted in a 30‐year aged spruce plantation nearby the Maoxian Mountain Ecosystem Research Station of the Chinese Academy of Sciences (31°42′N, 103°54′E) in the eastern Tibetan Plateau, China. The area has a montane temperate climate. The average annual temperature is 8.9°C, and the average annual precipitation is 900 mm, about 70% of which falls during May to September. The soil is classified as a calcic luvisols soil type according to the IUSS Working Group WRB (2007). It is a silt loam with 15.5% sand, 62.5% silt, and 21.9% clay. In 2008, the topsoil (0–10 cm) layer had a bulk density of 0.94 g/cm^3^, pH of 4.67 (H_2_O, at 1:2.5 w/v), total soil organic carbon concentration (SOC) of 43.6 g/kg, and soil total nitrogen (TN), total phosphorus (TP), and total potassium concentrations of 3.4 g/kg, 0.48 g/kg, and 18.1 g/kg, respectively (Jiang, Pang, & Bao, 2011).

### 2.2 Experimental design

In August 2008, we set up a thinning experiment with a randomized block design in a spruce plantation which was built in 1980s to investigate the potential effect of thinning on restoration of understory plant abundance, diversity, and associated ecosystems function which understory plants deliver. Three similar blocks were selected, and four thinning treatments including un‐thinning control (canopy stand, CK), small‐sized gap (about 74 m^2^, SG), medium‐sized gap (about 109 m^2^, MG), and large‐sized gap (about 196 m^2^, LG) were randomly arranged in each block in the winter of 2008. The designed size of artificial gaps was based on a prior investigation concerning the size of canopy gap occurring frequently in the forests without apparent disturbance due to human activities in temperate region (Tan, Zhu, Kang, & Zhang, 2007). To avoid confounding effects caused by soil disturbance due to the use of heavy machinery, gaps were created by felling trees using chainsaw in the center region of each plot. Specifically, 3, 5, and 12 spruce trees were felled in the SG, MG, and LG treatments, respectively; while no tree was felled in the CK plots. There was a buffering zone with more than 30 m for any neighboring plots. To exclude the possible random error due to gap shape on real acreage of openness and microclimate, plant and soil properties, a polygon about 400 m^2^ in size with similar shape based on field condition was marked before thinning. Once thinned, the stems, branches, and leaves of the harvested trees were removed, whereas understory shrubs, grasses, and herbs as well as the stumps at 50 cm above the ground were retained.

### 2.3 Plant cover investigating and soil sampling

Shrubs and herbs cover were investigated before soil sampling. To ensure accuracy, the quadrat in each plot was divided by sticks into 16 segments of equal area when estimating the vascular cover. Ten subsamples from top soil layer (0–10 cm) were randomly taken with a soil auger (Φ = 2.5 cm) along the two diagonal lines of 1 × 1 m quadrat established in the central part of each gap or control on 21st April 2014. Immediately after sampling, soil subsamples from each plot were pooled as a composite sample after they were mixed thoroughly, placed in separate zipper‐type plastic bag within an insulated box and transported to the laboratory. In the laboratory, the leaves, plant roots, and gravel within individual soil sample were eliminate using sieve of 2 mm. Next, the fine root samples were oven‐dried for 24 hr at 65°C and weighed. After being thoroughly mixed, each soil sample was divided into three parts for the determination of soil water content, soil microbial community, soil nematodes, and soil physicochemical properties. Soil water content was determined immediately after sample separation. The samples used to analyze soil nematodes and microbial community composition were kept at 4°C in a refrigerator until further analysis, and all samples were processed within 1 week. The samples used to analyze pH, SOC, and TN were air‐dried under room temperature.

### 2.4 Soil physicochemical properties analysis

Soil water content (% SWC) was determined by drying about 50 g fresh soil samples at 105°C for 48 hr. Other soil physicochemical properties were determined with air‐dried soil samples. Before analysis, soils were air‐dried for 14 days at room temperature and were ground and passed through a 0.25‐mm sieve. SOC and TN were determined using a Vario MACRO cube CHNS Elementary Analyzer (Elementar Analysensysteme GmbH, Hanau, Germany) at 850°C. Soil pH was determined with deionizered water to air‐dried and fine ground sample at the ratio of 1:2.5 (weight to volume, w/v) with an electronic pH meter.

### 2.5 Nematode extraction and identification

Nematodes were extracted from 100 g fresh soil samples using modified Baermann funnel (Ingham & Santo, 1994). The nematodes recovered were counted, heat killed at 60°C, fixed with triethanolamine formalin, transferred to flamed glass slides with approximately 1 ml of fixative, and observed using an inverted compound microscope. A total of 100 specimens per sample were randomly selected and identified to the genus following the keys for Nematoda by Bongers (1994) and Ahmad and Jairajpuri (2010), Siddiqi (2000) as well as the Interactive Diagnostic Key to Plant Parasitic, Free‐living and Predaceous Nematodes (https://nematode.unl.edu/nemakey.htm) at a 400× or 1000× magnification. If fewer than 100 nematodes were observed in one sample, all specimens were identified. Nematode abundance was adjusted according to soil moisture and was expressed as number of nematodes per 100 g dry soil. Then, soil nematodes were classified as plant parasites (Pp), bacterivores (Ba), fungivores (Fu), and omnivore–predators (OP), according to Yeates, Bongers, de Goede, Freckman, and Georgieva (1993). Nematode type was classified along the colonizer–persister (c–p) gradient as described by Bongers (1990), Bongers, Alkemade, and Yeates (1991), and Bongers and Bongers (1998). Meanwhile, each nematode taxon was also assigned to a functional guild defined using a combination of feeding group and life history traits expressed as c‐p scores ranging from 1 (r‐strategist) to 5 (K‐strategist) (Bongers, 1990; Bongers & Bongers, 1998).

The assumed size‐dependent effects of canopy gaps on soil nematodes were examined with the following variables: (1) total nematodes abundance; (2) abundance of individual trophic groups including Pp, Ba, Fu, OP; (3) Margalef richness index (Margalef, 1958); (4) Shannon–Weaver index (Shannon & Weaver, 1949); (5) Simpson's dominance index (Simpson, 1949); (6) Pielou's evenness index (Pielou, 1975); (7) Maturity index (MI) and (8) Plant‐parasitic index (PPI) (Bongers, 1990); (9) Nematode channel ratio (NCR = Ba/(Ba + Fu)), where Ba and Fu are abundances of bacterial feeders and fungal feeders, respectively; (10) Basal index (BI), Channel index (CI), Enrichment index (EI), and (11) Structure index (SI) (Ferris, Bongers, & de Goede, 2001); (12) the ratio of predators–omnivore to plant‐feeding nematodes (OP/Pp) (Eisenhauer, Migunova, Ackermann, Ruess, & Scheu, 2011).

### 2.6 Phospholipid fatty acid analysis

About 15 g of fresh soil from each sample was frozen in a refrigerator and was processed for assessment of fungal and bacterial fatty acid markers within one week after sampling. Phospholipid fatty acids (PLFAs) were extracted from freeze‐dried soil samples with reference to the method of Bossio and Scow (1998) and quantified by gas chromatography. The weights of individual PLFAs were measured as ng/g dry soil. Total microbial biomass was estimated from the total concentrations of all the PLFAs detected in samples. Bacterial biomass was estimated from the total concentrations the *i*14:0, *a*14:0, 14:0, *i*15:0, *a*15:0, 15:0, *i*16:0, 16:1ω5c, *i*17:0, *a*17:0, cy17:0, 17:0, and cy19:0, and fungi biomass was estimated from the summered concentrations of 18:1ω5c, 18:1ω9c, and 18:2ω6, 9c.

### 2.7 Data analysis

Effects of gap size on response variables such as abundance, relative abundance of nematode genera and/or trophic groups, morphological diversity, and functional diversity of soil nematodes were analyzed with a linear mixed model with the gap size as fixed effect and block as random effect. All mixed‐effect models were fitted to the data with the *lme* function included in the “*nlme”* package. When the effect of gap size on a given variable was significant, difference between groups was compared with a post hoc test using Tukey's honestly significant difference (HSD) tests at α = 0.05 level. Meanwhile, relationships between nematode attributes (including abundance, diversity, and specified indices) and environmental factors (such as fine root biomass, plant cover, soil physicochemical, and microbial properties) were examined with the Pearson correlation individually.

Subsequently, principal component analysis (PCA) was utilized to analyze how soil nematode communities were influenced by gap size. Nematode abundances were arsine square‐root transformed prior to performing PCA, because in most cases data of nematode counts are skewed. Rare species were not down‐weighted because they may represent taxa which are sensitive to thinning and/or play an important role in soil function. A Monte Carlo permutation option was employed to determine the significance of the first and second axes.

Finally, differences in taxonomic composition of soil nematode community (log (*x* + 1) transformed abundance data) across treatments were investigated with nonmetric multidimensional scaling (NMDS) with the square‐root transformed raw abundance of soil nematodes. The differences across treatments were tested with a multiresponse permutation procedure (MRPP) to evaluate whether sample units can be assigned to discrete groups (Mielke, 1991). In addition, statistical differences in community composition within canopy gaps of different sizes were assessed by permutational multivariate analysis of variance (Per‐MANOVA) based on 9999 restricted permutations of the data. The PC‐ORD 5.0 program (MjM Software, Gleneden Beach, OR, USA) was used to obtain the MRPP and per‐MANOVA.

## 3 RESULTS

### 3.1 Soil and plant properties

Soil bulk density, soil water content, pH, TOC, TN, fine root biomass, and litter depth were resistant to the effect of gap size (Table 1). However, the shrubs and herbs cover as well as soil microbial biomass were responsive to gap size (Table 1). In general, increase in gap size resulted in stimulated shrubs, grasses, and herbs cover as well as soil microbial biomass.

**Table 1:** Overview of main effect of gap size on environmental factors based on linear mixed models with gap size as fixed factor and block as random factor

Variable	CK	SG	MG	LG	*P*_GS_	*P*_B_
FRM (g)	1.96 ± 0.14	1.37 ± 0.48	2.14 ± 0.53	0.77 ± 0.17	.22	.23
LD (cm)	3.83 ± 0.88	2.83 ± 0.33	3.67 ± 0.44	3.87 ± 0.28	.57	.78
BD (mg/cm^3^)	96.67 ± 2.03	96.40 ± 1.80	94.03 ± 2.64	99.04 ± 1.81	.73	.37
SWC	34.0 ± 1.0	37.0 ± 1.0	35.0 ± 1.0	36.0 ± 1.0	.33	.75
pH	5.07 ± 0.16	4.94 ± 0.11	5.01 ± 0.06	4.90 ± 0.12	.64	.61
SOC	49.0 ± 3.8	52.7 ± 3.7	48.0 ± 2.4	47.7 ± 4.2	.52	.36
TN	4.0 ± 0.4	4.4 ± 0.3	4.1 ± 0.2	4.1 ± 0.2	.57	.52
T‐PLFA	42.13 ± 2.30^ab^	34.45 ± 4.36^b^	43.48 ± 5.13^ab^	52.59 ± 8.02^a^	.01	.12
SC (%)	7.7 ± 1.5^c^	20.3 ± 3.2^bc^	26.7 ± 3.5^b^	48.0 ± 6.8^a^	.04	.11
H&GC (%)	13.7 ± 2.3^c^	38.7 ± 8.1^b^	57.7 ± 8.4^a^	47.2 ± 4.8^ab^	.03	.07

FRM (g), fine root biomass (g); LD (cm), litter depth (cm); BD, soil bulk density (mg/cm^3^); SWC, soil water content (%); SOC, total soil organic carbon concentration (g/kg); TN, soil total nitrogen concentration (g/kg); T‐PLFA, total biomass of phospholipid fatty acids (ng/g); SC, shrubs cover (%); HC&GC, Herbs & grasses cover (%)***.*** Different lowercase letters represent difference across gaps was significant at α = 0.05 using Tukey's honestly significant difference tests. *P*_GS_ and *P*_B_ represent the *p*‐values of gap size effect and block effect based on linear mixed model with gap size as fixed factor and block as random factor, respectively. CK, control; SG, small gap; MG, medium gap; LG, large gap.

### 3.2 Nematodes density

**Figure 1:**
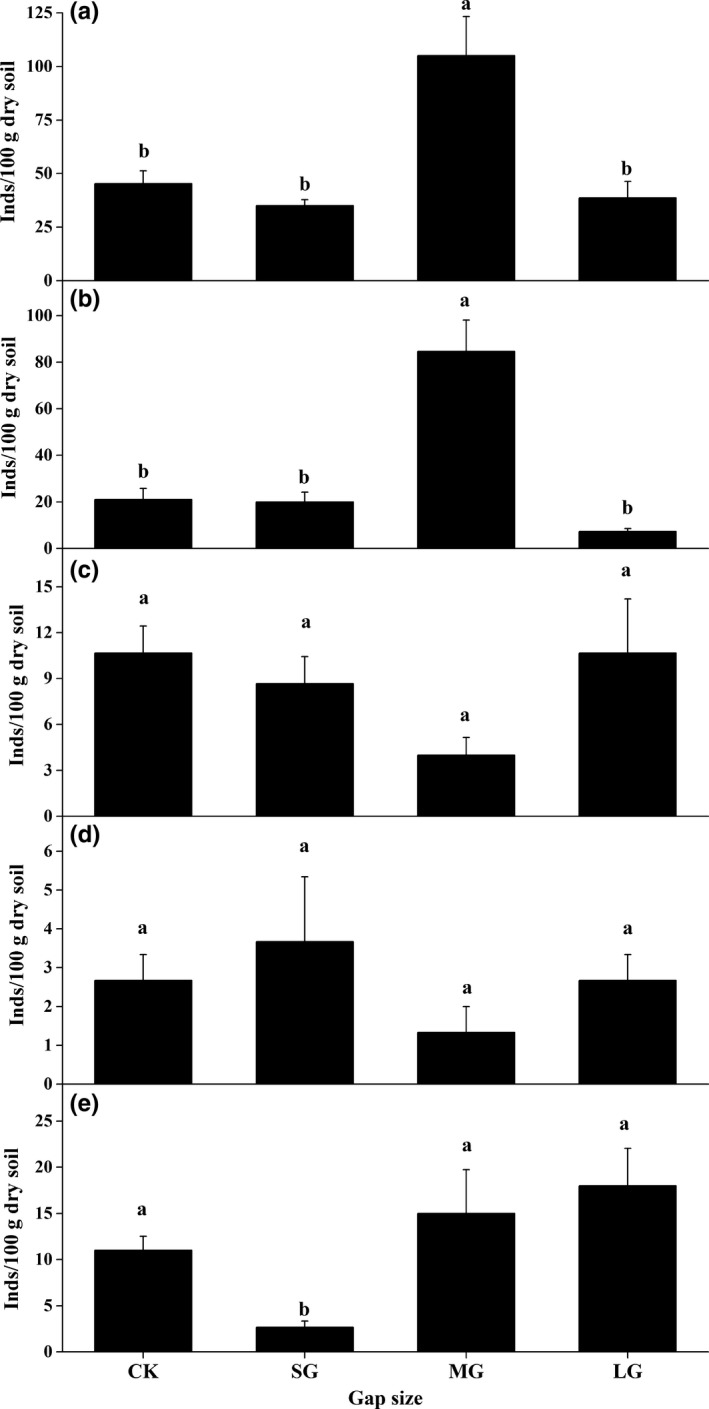
Mean abundance (±*SE*,* N* = 3) of total nematodes (a), plant parasites (b), bacterivores (c), fungi feeders (d), and predators–omnivore (e) under different‐sized gaps of spruce plantations. CK, SG, MG, and LG represent closed forests, small gaps, medium gaps, and large gaps, respectively . Means with the same letter are not different at the 5% level of significance based on the Tukey’s honestly significant difference tests.

The effect size of openness on soil nematode abundance was dependent more on the feeding habits of soil nematodes than block (Table 2). In general, gap size resulted in demonstrable changes in total nematodes abundance (Figure 1a), plant parasites abundance (Figure 1b), and predators–omnivore abundance (Figure 1e), while subtle changes were observed in the abundances of bacterivores (Figure 1c) and fungivores (Figure 1d). Specifically, total nematode abundance (Figure 1a) and plant parasites abundance (Figure 1b) significantly increased in the MG, while the abundance of predators–omnivore significantly reduced in the SG (Figure 1e). The abundance of total nematodes and that of free‐living nematodes was negatively correlated with soil bulk density, whereas the abundance of omnivore–predators was negatively correlated with soil bulk density and shrubs cover, respectively (Table 3).

**Table 2:** Overview of main effect of gap size on the abundances of trophic groups and total nematodes in spruce plantation based on linear mixed models with gap size as fixed factor and block as random factor

Effect	*df*	Total		Pp		Ba		Fu		OP	
		*F*	*p*	*F*	*p*	*F*	*p*	*F*	*p*	*F*	*p*
GS	3	78.10	.0005	2.44	.2401	2.15	.2365	4.50	.0900	21.09	.0065
B	2	5.88	.0644	1.35	.3574	1.79	.2785	1.22	.3868	2.00	.2500

GS, gap size; B, block. Ba, bacterivores; Fu, fungivores; Pp, plant parasites and OP, omnivores & predators.

**Table 3:** Relationships between Ln(*x* + 1) transformed nematode abundances and environmental factors based on Pearson correlation

Factor	Total		PP		Ba		Fu		OP		FFR	
	*r*	*p*	*r*	*p*	*r*	*p*	*r*	*p*	*r*	*p*	*r*	*p*
FRM	.37	.12	.34	.14	.38	.11	−.32	.15	.3	.17	.35	.13
BD	−.54	.03	−.25	.22	−.46	.07	.04	.45	−.66	.01	−.65	.01
SWC	−.18	.29	0	.5	−.38	.11	.14	.34	−.18	.29	−.3	.17
pH	.07	.42	.07	.42	.11	.36	−.17	.3	.06	.43	.1	.38
SOC	.12	.35	.13	.35	−.09	.39	.1	.38	.08	.41	.01	.49
TN	.2	.27	.3	.17	−.25	.22	.38	.11	.38	.11	.09	.39
T‐PLFA	.06	.43	.19	.27	.14	.33	−.16	.31	−.33	.15	−.05	.43
SC	−.17	.29	.07	.41	−.07	.41	−.01	.48	−.69	.01	−.35	.13
H&GC	.43	.08	.41	.09	.49	.05	−.18	.29	0	.49	.36	.12

FRM (g), fine root biomass (g); BD, soil bulk density (mg/cm^3^); SWC, soil water content (%); SOC, total soil organic carbon concentration (g/kg); TN, soil total nitrogen concentration (g/kg); T‐PLFA, total biomass of phospholipid fatty acids (ng/g); SC, shrubs cover (%); HC&GC, Herbs & grasses cover (%)***.*** Ba, bacterivores; Fu, fungivores; FFR, free‐living nematodes; Pp, plant parasites and OP, omnivores & predators.

### 3.3 Nematode diversity

**Figure 2:**
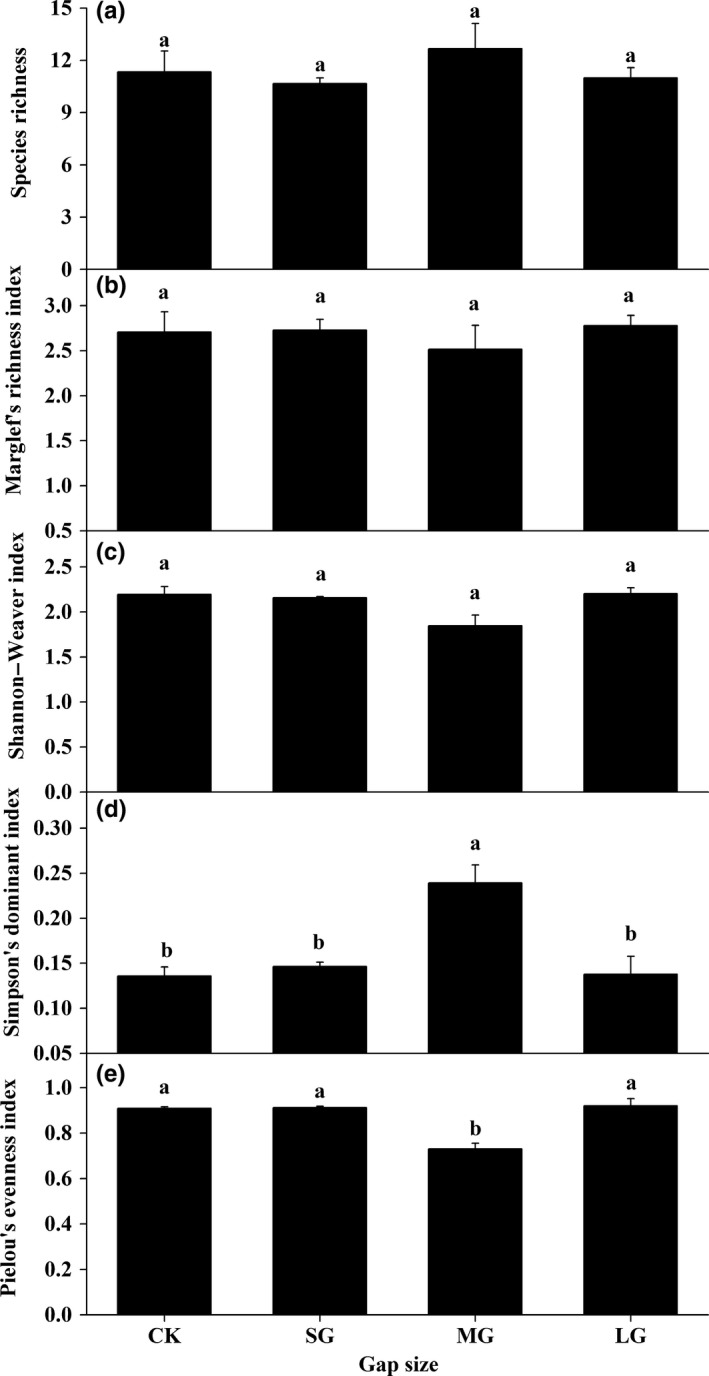
Mean diversity indices (±*SE*,* N* = 3) characterizing soil nematode communities under different‐sized gaps of spruce plantations. (a) Species richness; (b) Margalef's richness index; (c) Shannon–Weaver diversity index; (d) Simpson's dominance index; (e) Pielou's evenness index. CK, SG, MG, and LG represent closed forests, small gaps, medium gaps, and large gaps, respectively

The block did not affect any diversity index of soil nematodes, while the effect of gap size showed a great variation among the variables charactering the diversity of soil nematodes (Table S2). Generally, the genus richness (Figure 2a), Margalef's richness index (Figure 2b), and Shannon–Weaver index (Figure 2c) were resistant to change in gap size, while Simpson's dominant index (Figure 2d) and Pielou's evenness index (Figure 2e) were responsive to gap size. Specifically, the Pielou's evenness index of the MG was significantly lower than that of CK, SG, and LG, while there was no significant difference among the later three treatments, and the opposite trend was true for the Simpson's dominant index.

### 3.4 Faunal indices and the ratio of trophic groups

The Sigma MI, MI, MI_2–5,_ BI, EI, and SI were sensitive to the change of gap size (*p* < .05) but not to block (*P* > .05), while the PPI, CI, and NCR were consistently insensitive to the gap size and block (Table 4). The PP/OP ratio was significantly affected by the gap size (*F* = 10.53, *df* = 3, *p* = .02) but not block (*F* = 3.71, *df* = 2, *p* = .12).

**Table 4:** Overview of main effect of gap size on functional diversity indices of soil nematodes in spruce plantation based on linear mixed models with gap size as fixed factor and block as random factor

Variable	CK	SG	MG	LG	*P*_GS_	*P*_B_
∑MI	2.62 ± 0.02^ab^	2.77 ± 0.06^a^	2.48 ± 0.05^b^	2.37 ± 0.14^b^	.02	.07
MI	2.82 ± 0.15^ab^	3.07 ± 0.12^a^	2.57 ± 0.02^b^	2.23 ± 0.08^b^	.001	.07
MI2‐5	2.82 ± 0.15^ab^	3.22 ± 0.06^a^	2.59 ± 0.01^b^	2.53 ± 0.07^b^	.005	.46
PPI	2.32 ± 0.18	2.09 ± 0.09	2.34 ± 0.12	2.50 ± 0.19	.39	.50
BI	26.30 ± 5.86	11.18 ± 0.78	36.12 ± 0.78	25.94 ± 5.18	.04	.92
CI	100.00 ± 0.00	43.70 ± 28.27	66.67 ± 33.33	19.32 ± 0.68	.24	.99
EI	14.04 ± 4.34^b^	47.01 ± 12.4^ab^	8.88 ± 4.89^b^	56.56 ± 10.99^a^	.03	.62
SI	72.73 ± 5.94^ab^	87.14 ± 1.44^a^	62.47 ± 0.69^b^	60.02 ± 2.76^b^	.005	.51
NCR	0.84 ± 0.05	0.81 ± 0.04	0.97 ± 0.01	0.79 ± 0.08	.24	.92

Different lowercase letters in the same column represent a significant difference across treatments (α = 0.05) using Tukey's Honestly Significant Difference tests. MI, maturity index; PPI, plant‐parasitic index; NCR, nematode channel ratio; EI, enrichment index; and SI, structure index. CK, control; SG, small gap; MG, medium gap; LG, large gap.

### 3.5 Community similarity of soil nematodes

A total of 30 genera of soil nematodes, including 11 genera plant parasites, 10 bacterivores, eight omnivore–predators, and one fungivores were found in this study. At the level of genus, contrasting soil nematode communities were collected from canopy gaps of different sizes (Table S3). In the CK, the dominant genera were the *Plectus*,* Filenchus*,* Heterodera,* and *Clarkus*. In the SG, the dominant genera were the *Filenchus*,* Heterodera*,* Nagelus,* and *Aphelenchus*. Nematodes in the MG were dominated by the *Heterodera* and *Filenchus,* whereas the nematodes in the LG were dominated by the *Eudorylaimus*,* Mesodorylaimus,* and *Plectus*. At the level of guilds, different gap size yielded distinct nematodes (Tables S4 and S5). In the CK, the dominant guilds were the plant parasite of cp‐2 & cp‐3, bacterivores of cp‐2, and predators–omnivore of cp‐4 guilds. In the SG, the dominant guilds were plant parasites of cp‐2, cp‐3, bacterivore of cp‐2, and fungivore of cp‐2. In the MG, the dominant guilds were plant parasites of cp‐2 and cp‐3, whereas in the LG, the dominant guilds were predators–omnivores of cp‐4 and cp‐5 as well as plant parasite of cp‐2.

**Figure 3:**
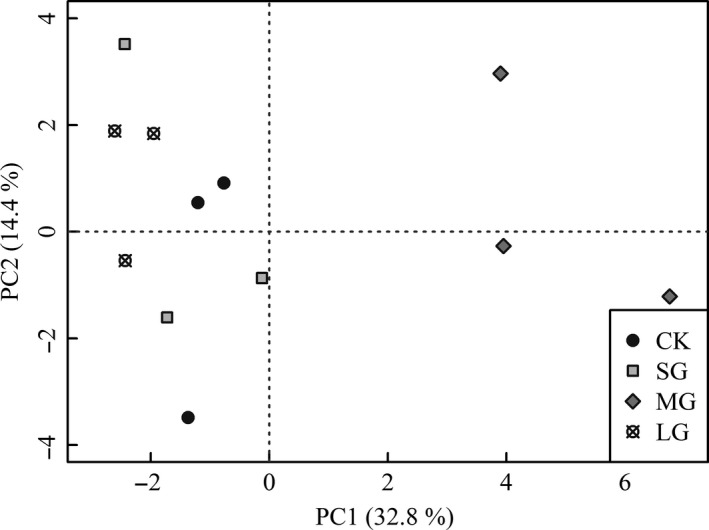
Ordination diagram of principal component analysis (PCA) for soil nematode communities under different‐sized gaps in spruce plantations. Percentages of total explained variation by PCA axes are given in parentheses. CK, SG, MG, and LG represent closed forests, small gaps, medium gaps, and large gaps, respectively

**Figure 4:**
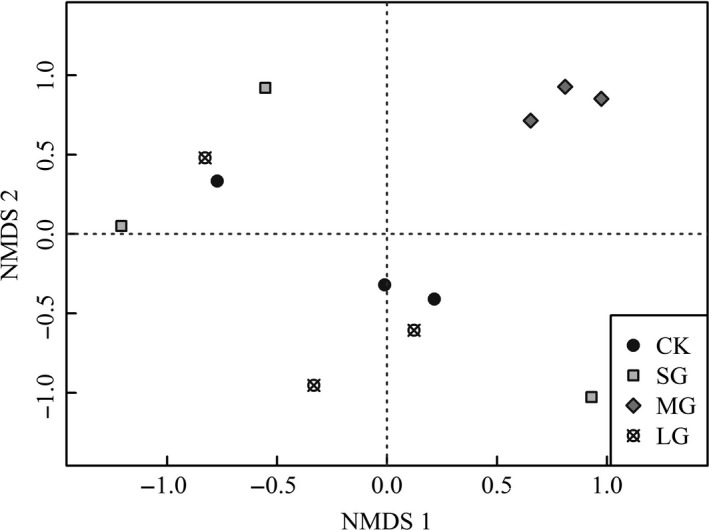
The ordination plot of nonmetric multidimensional scaling (NMDS) for soil nematode community under distinct‐sized canopy gaps of spruce plantation. Data were transformed using log(*x* + 1) prior to analysis, and the ordination was based on three pooled samples per treatment and 32 genera (*final stress* = 9.85, *final instability* = 0.00, *p* = .004). CK, SG, MG, and LG represent closed forests, small gaps, medium gaps, and large gaps, respectively

The first four principal components (PCs) accounted for 69.2% of the variation for the community composition of soil nematodes across canopy gap regimes. The PC1 and PC2 accounted for 32.8% and 14.4%, respectively (Figure 3). NMDS analysis shown that gap size resulted in contrasting soil nematode communities in the spruce plantation (Figure 4, *final stress* = 9.85, *final instability* = 0.00, *p* < .01). Per‐MANOV analysis suggested that soil nematode communities showed a considerable variation across the gap sizes tested (*F* = 2.11, *df* = 3, *p* < .01). The MRPP analysis also revealed that the gap size resulted a considerable effect on soil nematode community (*T* = −2.84, *A* =  0.33, *p* < .01). Specifically, the soil nematode community of MG was significantly distinct from that of the CK, SG, and LG, while the dissimilarity of the soil nematode communities of latter three treatments was not statistically significant (Table 5).

**Table 5:** Pairwise comparisons for soil nematodes under different‐sized canopy gaps in spruce plantations using the multiresponse permutation procedures based on Sorensen (Bray–Curtis) distance

Group compared	*T*	*A*	*P*
CK vs. SG	0.19	−0.02	.49
CK vs. MG	−2.75	0.40	.02
CK vs. LG	0.16	−0.02	.53
SG vs. MG	1.95	0.19	.04
SG vs. LG	−0.45	0.05	.30
MG vs. LG	−2.82	0.43	.02

A, the chance‐corrected within‐group agreement, $A=1-(\delta /m_{\delta })$; *T* is a one to one function of $m_{\delta }$, $T=\left(\delta -m_{\delta }\right)/s_{\delta }$, $\delta =\sum_{i=1}^{g}C_{i}x_{i}$, where *C* is a weight that depends on the number of items in the groups (normally *C*_*i*_ = *n*_*i*_/*N*, where *n*_*i*_ is the number of items in group *i* and *N* is the total number of items, *x*_*i*_ is the average distance within each group *i,* m_δ_ and s_δ_ are the mean and standard deviation of δ under the null hypothesis; and *P*, the probability of randomly getting a smaller distance than the average distances for the true groups. CK, control; SG, small gap; MG, medium gap; LG, large gap.

## 4 DISCUSSION

Numerous studies have demonstrated the effects of canopy openings resulting from natural disturbance such as windstorm, insect‐attacks, and/or stand‐replacing fires on soil nematode community (e.g., Butenko, Gongalsky, Korobushkin, Ekschmitt, & Zaitsev, 2017; Čerevková & Renčo, 2009; Čerevková, Renčo, & Cagáň, 2013; Renčo, Čerevková, Homolová, & Gömöryová, 2015; Sohlenius, 1996, 1997, 2002). High input of dying plants and roots can stimulate soil organism activity for several years after the cutting (Sohlenius, 1996, 1997, 2002). We predicted that soil nematodes would respond less to thinning in comparison with these disturbances because the magnitude of soil disturbance due to windstorm, beetle‐attack, and stand‐replacing fires on microclimate, soil biogeochemistry, plant characteristics, and soil microbial community is expected to be drastically than that of artificial thinning. Therefore, we investigated that the responses of soil nematode community to contrasting thinning events in this study.

### 4.1 Nematodes abundance

In agreement with our first two hypotheses, the effects of openness on soil nematodes abundance vary with gap size and feeding group of nematodes (Figure 1). Collectively, soil nematode abundance was not linearly correlated with gap size. Meanwhile, the plant parasites and predator‐omnivores were found to be more susceptible than bacterivores and fungivores. To be specific, the maximal abundance of plant parasites occurred in the medium gaps, whereas the minimal abundance of omnivore–predators occurred in the small gaps (Figure 1). One study revealed that the population size of decomposers, particularly nematodes in gaps of a seminatural mixed beech and ash forests in Northern Europe was generally stimulated in comparison with closed canopies in the early stage of litter decomposition (Bjørnlund & Christensen, 2005). Another study demonstrated the contrasting seasonal responses of soil nematodes during the first three years after gap formation in a beech‐dominated forest under seminatural condition (Ritter & Bjørnlund, 2005). However, whether or how gap formation would affect soil nematodes in the long run has not been explored. Herein, this knowledge gap was filled by comparing soil nematode community in contrasting‐sized gaps to closed canopy after 6 years of implementing structured thinning.

A key finding was that both total nematodes abundance and free‐living nematodes were negatively correlated with soil bulk density; predators–omnivores abundance was negatively correlated with soil bulk density and shrubs cover (Table 3). Other study has suggested that abundance of herbivorous nematodes is positively correlated with fine root mass (Bonwman and Arts 2000; van Eekeren *at al*. 2009). In our study phytophagous nematodes were greatly affected by plant community characteristics (Yeates, 1999), and the fine root mass is a proxy of plant community characteristics. Plants are expected to affect soil organisms directly and/or indirectly through changing microclimate, quality, and quantity of litter and root exudates as well as the interactions among components of soil food webs. Our results are inconsistent with another study which found that the responses of soil nematodes were possibly regulated by carbon inputs through living roots (Ritter & Bjørnlund, 2005). The predators–omnivores abundance and environmental factors including soil bulk density and shrubs cover (Table 3), this is contrast to the notion that microbial production and the moisture contents were important factor affect animal abundance (Sohlenius, 1982).

The high sensitivity of predators and omnivores in comparison with microbivorous nematodes is in line with the proposal that higher trophic levels are more vulnerable to environmental change than lower trophic levels (Cesarz et al., 2017; Hines, Eisenhauer, & Drake, 2015; Voigt, Perner, & Hefin Jones, 2007). One likely reason is that soil biota in the higher trophic level needs longer time to accommodate changes in habitat factors than that in lower trophic level due to the life strategy (Ferris et al., 2001; Valladares, Cagnolo, & Salvo, 2012); another possible explanation may be the distinct linkages among guilds in the soil food webs, because different trophic levels participate in differential interactions, including competition, predation, and mutualistic symbiosis.

### 4.2 Diversity and ecological indices

In this study, the effect of gap size on taxonomic richness (Figure 2a), Margalef's richness index (Figure 2b), and Shannon–Weaver index (Figure 2c) was not significant. However, the effects of gap size on Simpson's dominant index (Figure 2d) and Pielou's evenness index were complicated. Specifically, the minimal Pielou's evenness occurred in the medium gaps (Figure 2e), indicating soil nematode community in medium gaps was dominated by fewer genera in comparison with other treatments. If the taxa richness for two communities is identical, the community with a higher evenness contributes more to the maintenance of functional stability than that with lower evenness when the same ecosystem was exposed to environmental stresses (Wittebolle et al., 2009). Hence, the soil nematode community in medium gap of spruce plantation in our study is vulnerable to disturbance.

Structure index, which is commonly used to reflect soil food web connection and length, was significantly affected by gap size and site heterogeneity. Furthermore, gap size exerted demonstrable effects on the density ratio of predators–omnivore to plant parasites (OP/Pp), a variable to indicate food web complexity concerning the potential combined predation ability of omnivorous and carnivorous nematodes to herbivorous nematodes (Eisenhauer et al., 2011), implying that gap formation would affect the links between these two trophic groups.

The maturity index of nematodes is one of the key indices of soil health, and also an indication of disturbance. In our study, ∑MI, MI, and MI increased with the gap size (Table 4), indicating that thinning result in a lower MI value. This result is in agreement with the proposal that disturbance generally leads to a lower MI (Bongers, 1990). In contrast, Sohlenius (1997, 2002) found that clear‐cutting supporting a more mature nematode fauna characterized by a higher MI value than uncut pine forests. Besides, thinning did not affect PPI, and this result is line with other studies which suggest that there no significant effect of disturbance due to fire or windstorm on PPI (Čerevková & Renčo, 2009; Renčo et al., 2015).

### 4.3 Community structure

Evidence supported our hypothesis that gap size exerted significant effects on soil nematodes community composition (Tables 4, 5 and S3–S5; Figures 3 and 4). In contrast to the proposal that soil nematodes are not susceptible to change in canopy openness, herb cover, and litter depth (Matlack, 2001), we found a contrasting soil nematode community characterized by higher abundance of *Filenchus* and *Heterodera* in medium gaps in comparison with other treatments. One likely reason for our finding may be correlated with the plant–soil feedbacks resulting from the changes in microclimate, soil physicochemical properties, plant including cover, and diversity as well as soil microbial community. After all, abundance of *Filenchus* and diversity of mycorrhizal fungi are closely correlated (Hánel, 1996).

## 5 CONCLUSIONS

In conclusion, thinning‐induced gap size significantly changed abundances of plant parasites and omnivores‐predators, whereas it did not affect microbial‐feeding nematodes (Table 2, Figure 1). Additionally, two evidences support that differential gap sizes resulted in contrasting nematode assemblages in soils of a spruce plantation. On one hand, gap creation altered soil nematodes diversity (Figure 2) and most of the nematode weighted diversity indices (Table 4); on the other hand, medium‐sized gaps supported a nematode community with distinct structure and composition compared with other gaps and control (Tables 5 and S3–S5; Figures 3 and 4). One limitation of our study lies in that the results are based on one sampling time point in a local site without a baseline data. Although we found evident effects of gap size on nematode attributes, long‐term dynamics of nematodes over distinct environmental conditions in a larger scale with more tree species is still needed to draw robust and generalized conclusion on thinning on soil food webs.

## CONFLICT OF INTEREST

None declared.

## AUTHOR CONTRIBUTION

XYP and WKB designed the experiment. BY conducted the experiment and wrote the draft. KXZ, XYP, and WKB discussed and revised the draft.
