# Supplementary material for: Thinning‐induced canopy opening exerted a specific effect on soil nematode community
Source: Ecol Evol. 2018 Mar 14;8(8):3851–61. doi: 10.1002/ece3.3901 (PMC5916288; doi:10.1002/ece3.3901)
Supplement: Supplementary file 1 [file ECE3-8-3851-s001.doc]

**Table S_1_**. Major soil physiochemical properties and microbial biomass parameters, including microbial biomass carbon (MBC), microbial biomass nitrogen and microbial biomass phosphorus (MBP) (Mean ± SE, *N*=3) in different-sized openness of spruce plantation in the eastern Tibetan Plateau, China in the winter of 2008

| Variable | CK | SG | MG | LG | P_GS_ | P_B_ |
| --- | --- | --- | --- | --- | --- | --- |
| Bulk density (g/cm^3^) | 0.92 ± 0.05 | 0.98 ± 0.04 | 0.90 ± 0.04 | 0.96 ± 0.03 | 0.61 | 0.68 |
| SWC (%） | 32.0 ± 1.0 | 39.0 ± 2.0 | 37.0 ± 5.0 | 38.0 ± 2.0 | 0.27 | 0.47 |
| MBC (mg/kg) | 613.04 ± 46.26 | 641.89 ± 176.40 | 573.20 ± 34.95 | 607.46 ± 31.67 | 0.28 | 0.03 |
| MBN (mg/kg) | 89.45 ± 7.21 | 103.31 ± 39.21 | 98.28 ± 12.82 | 83.15 ± 5.54 | 0.26 | 0.04 |
| MBP (mg/kg) | 15.01 ± 2.62 | 9.21 ± 2.00 | 11.84 ± 3.37 | 8.51 ± 1.26 | 0.85 | 0.67 |
| NH_4_^+^-N (mg/kg) | 21.42 ± 3.32 | 20.75 ± 1.01 | 20.13 ± 3.48 | 15.18 ± 0.26 | 0.47 | 0.74 |
| NO_3_^-^-N (mg/kg) | 7.29 ± 1.94 | 16.16 ± 6.24 | 8.42 ± 2.33 | 15.97 ± 4.60 | 0.11 | 0.12 |
| DOC (mg/kg) | 120.18 ± 13.27 | 133.21 ± 21.48 | 133.15 ± 33.77 | 112.22 ± 9.30 | 0.53 | 0.49 |
| DON (mg/kg) | 8.75 ± 1.26 | 17.51 ± 6.02 | 10.19 ± 2.72 | 15.09 ± 2.71 | 0.05 | 0.07 |
| pH (H_2_O at 1:2.5 w/v) | 4.83 ± 0.27 | 4.45 ± 0.11 | 4.90 ± 0.02 | 4.48 ± 0.23 | 0.58 | 0.90 |
| TN (g/kg) | 3.29 ± 0.04 | 3.70 ± 0.17 | 3.48 ± 0.14 | 3.76 ± 0.19 | 0.12 | 0.27 |
| TP (mg/kg) | 411.66 ± 37.59 | 414.48 ± 14.89 | 434.07 ± 16.32 | 364.91 ± 85.25 | 0.29 | 0.32 |
| K (g/kg) | 18.62 ± 0.21 | 17.68 ± 1.42 | 18.21 ± 0.29 | 17.73 ± 0.63 | 0.05 | 0.02 |
| TOC (g/kg) | 42.70 ± 1.40 | 42.08 ± 1.84 | 43.05 ± 1.85 | 46.89 ± 1.96 | 0.12 | 0.24 |
| CEC (mmol/kg) | 267.19 ± 11.57 | 269.58 ± 9.74 | 258.98 ± 6.83 | 262.74 ± 11.9 | 0.73 | 0.38 |
| Sand (%) | 18.45 ± 2.11 | 16.21 ± 4.96 | 13.21 ± 2.13 | 14.23 ± 1.58 | 0.45 | 0.06 |
| Silt (%) | 60.35 ± 2.39 | 61.21 ± 4.14 | 64.90 ± 1.1 9 | 63.62 ± 1.39 | 0.29 | 0.13 |
| Clay (%) | 21.20 ± 1.3.9 | 22.59 ± 1.99 | 21.89 ± 3.04 | 22.11 ± 1.58 | 0.94 | 0.39 |

Note: CK: control;; LG: large gap; MG: medium gap; SG: small gap;. Density: bulk density; SWC: soil water content; NH_4_^+^-N: soil ammonium nitrogen concentration; NO_3_^-^-N: soil nitrate nitrogen concentration; DOC: dissolved organic carbon; DON: dissolved organic nitrogen; TOC: total carbon concentration; TN: soil total nitrogen concentration; TP: soil total phosphorus concentration; K, soil potassium concentrations; CEC: Cation Exchange Capacity. *P_GS_* and *P_B_* represent the *P*-values of gap size effect and block effect based on linear mixed models with gap size as fixed factor and block as random factor, respectively.

***Table S_2_****. F- and P-values for linear mixed models with gap size as fixed factor and block as random factor on genera richness (S), Margalef richness index (SR), Shannon-Weaver index(H'), Simpson’s dominance index (λ) and Pielou’s evenness index(J') of soil nematode community in spruce plantation of differential sized openness*

| Effect | df | *S* | | *SR* | | *H'* | | *λ* | | *J'* | |
| --- | --- | --- | --- | --- | --- | --- | --- | --- | --- | --- | --- |
|  |  | *F* | *P* | *F* | *P* | *F* | *P* | *F* | *P* | *F* | *P* |
| GS | 3,4 | 0.52 | 0.68 | 0.75 | 0.56 | 4.29 | 0.06 | 11.09 | 0.007 | 20.20 | 0.002 |
| B | 2,4 | 0.72 | 0.53 | 0.14 | 0. 87 | 0.01 | 1.00 | 0.46 | 0.65 | 3.32 | 0.11 |

Note: GS, gap size; B, block.

**Table S_3_**. Abundance (Mean ± SE, *N* = 3, individuals/100 g dry soil) and relative abundance (Mean ± SE, *N* = 3, %) of nematode taxa in different-sized gaps of spruce plantation in the eastern Tibetan Plateau, China

| Group | Genus | CK | | SG | | MG | | LG | |
| --- | --- | --- | --- | --- | --- | --- | --- | --- | --- |
|  |  | D | RD | D | RD | D | RD | D | RD |
| PP2 | *Tylenchus* | 0 | 0 | 0.67 ± 0.67 | 1.90 ± 1.90 | 3.00 ± 1.53 | 2.74 ± 1.55 | 0.67 ± 0.67 | 1.59 ± 1.59 |
| PP2 | *Filenchus* | 6.00 ± 3.06 | 14.02 ± 7.16 | 4.00 ± 2.00 | 11.51 ± 5.70 | 23.33 ± 4.33 | 23.13 ± 5.20 | 3.67 ± 2.03 | 8.22 ± 4.81 |
| PP2 | *Cephalenchus* | 4.67 ± 3.71 | 8.80 ± 6.82 | 0.67 ± 0.67 | 2.22 ± 2.22 | 0.67 ± 0.67 | 0.47 ± 0.47 | 0 | 0 |
| PP2 | *Aglenchus* | 0.67 ± 0.67 | 1.39 ± 1.39 | 0.67 ± 0.67 | 1.67 ± 1.67 | 0 | 0 | 0.67 ± 0.67 | 1.33 ± 1.33 |
| PP2 | *Bastiania* | 0.67 ± 0.67 | 1.23 ± 1.23 | 0 | 0 | 0 | 0 | 0 | 0 |
| PP3 | *Criconemoides* | 4.00 ± 2.00 | 8.75 ± 4.01 | 0.67 ± 0.67 | 2.22 ± 2.22 | 7.33 ± 4.37 | 6.19 ± 2.71 | 0.67 ± 0.67 | 2.78 ± 2.78 |
| PP3 | *Paratylenchus* | 0 | 0 | 0.67 ± 0.67 | 1.90 ± 1.90 | 0.67 ± 0.67 | 0.84 ± 0.84 | 0 | 0 |
| PP3 | *Helicotylenchus* | 0 | 0 | 2.33 ± 2.33 | 5.83 ± 5.83 | 4.00 ± 2.31 | 4.24 ± 2.94 | 0 | 0 |
| PP3 | *Heterodera* | 5.00 ± 3.00 | 10.83 ± 6.07 | 4.67 ± 2.91 | 14.44 ± 9.88 | 42.33 ± 8.95 | 39.84 ± 1.36 | 1.67 ± 0.88 | 5.50 ± 3.69 |
| PP3 | *Nagelus* | 0 | 0 | 4.33 ± 3.38 | 11.39 ± 8.28 | 0 | 0 | 0 | 0 |
| PP3 | *Tylenchorhynchus* | 0 | 0 | 0.67 ± 0.67 | 1.67 ± 1.67 | 0.67 ± 0.67 | 0.82 ± 0.82 | 0 | 0 |
| PP3 | *Meloidogyne* | 0 | 0 | 0 | 0 | 0.67 ± 0.67 | 0.72 ± 0.72 | 0 | 0 |
| Ba1 | *Monhystera* | 1.33 ± 1.33 | 2.78 ± 2.78 | 0 | 0 | 0.67 ± 0.67 | 0.82 ± 0.82 | 1.33 ± 0.67 | 2.92 ± 1.48 |
| Ba1 | *Rhabditis* | 0 | 0 | 2.33 ± 1.45 | 6.98 ± 4.13 | 0.67 ± 0.67 | 0.82 ± 0.82 | 0.67 ± 0.67 | 1.59 ± 1.59 |
| Ba2 | *Plectus* | 6.00 ± 1.15 | 14.48 ± 4.76 | 1.33 ± 1.33 | 3.33 ± 3.33 | 0.67 ± 0.67 | 0.47 ± 0.47 | 4.00 ± 1.15 | 10.21 ± 2.04 |
| Ba2 | *Anaplectus* | 1.33 ± 0.67 | 3.20 ± 1.72 | 0.67 ± 0.67 | 2.22 ± 2.22 | 0.67 ± 0.67 | 0.82 ± 0.82 | 1.33 ± 0.67 | 2.92 ± 1.48 |
| Ba2 | *Panagrolaimus* | 0 | 0 | 1.67 ± 0.88 | 4.52 ± 2.49 | 0 | 0 | 2.00 ± 1.15 | 4.25 ± 2.32 |
| Ba2 | *Eucephalobus* | 0 | 0 | 0.67 ± 0.67 | 1.90 ± 1.90 | 0 | 0 | 0 | 0 |
| Ba2 | *Cephalobus* | 1.33 ± 0.67 | 2.62 ± 1.32 | 0.67 ± 0.67 | 1.90 ± 1.90 | 0 | 0 | 0 | 0 |
| Ba3 | *Chronogaster* | 0.67 ± 0.67 | 1.39 ± 1.39 | 0.67 ± 0.67 | 1.67 ± 1.67 | 0.67 ± 0.67 | 0.47 ± 0.47 | 1.33 ± 0.67 | 4.37 ± 2.41 |
| Ba3 | *Prismatolaimus* | 0 | 0 | 0.67 ± 0.67 | 2.22 ± 2.22 | 0.67 ± 0.67 | 0.72 ± 0.72 | 0 | 0 |
| Fu2 | *Aphelenchus* | 2.67 ± 0.67 | 5.82 ± 0.94 | 3.67 ± 1.67 | 10.56 ± 4.75 | 1.33 ± 0.67 | 1.19 ± 0.63 | 2.67 ± 0.67 | 7.03 ± 1.14 |
| OP4 | *Epidorylaimus* | 1.33 ± 0.67 | 3.20 ± 1.72 | 0.67 ± 0.67 | 1.90 ± 1.90 | 3.67 ± 2.73 | 3.70 ± 3.02 | 2.67 ± 0.67 | 7.29 ± 1.68 |
| OP4 | *Clarkus* | 4.33 ± 1.45 | 10.02 ± 3.26 | 0.67 ± 0.67 | 1.67 ± 1.67 | 1.33 ± 0.67 | 1.56 ± 0.79 | 1.33 ± 0.67 | 4.37 ± 2.41 |
| OP5 | *Mononchus* | 0 | 0 | 0 | 0 | 0.67 ± 0.67 | 1.30 ± 0.72 | 0 | 0 |
| OP4 | *Mylonchulus* | 0 | 0 | 0 | 0 | 0.67± 0.67 | 0.47± 0.47 | 0.67 ± 0.67 | 2.78 ± 2.78 |
| OP4 | *Coomansus* | 0 | 0 | 0.67 ± 0.67 | 2.22 ± 2.22 | 1.33 ± 1.33 | 0.95 ± 0.95 | 0 | 0 |
| OP4 | *Eudorylaimus* | 0.67 ± 0.67 | 1.39 ± 1.39 | 0.67 ± 0.67 | 2.22 ± 2.22 | 0.67 ± 0.67 | 0.47 ± 0.47 | 7.67 ± 5.36 | 18.94 ± 10.44 |
| OP4 | *Laimydorus* | 0.67 ± 0.67 | 1.23 ± 1.23 | 0 | 0 | 0.67 ± 0.67 | 0.72 ± 0.72 | 0 | 0 |
| OP5 | *Mesodorylaimus* | 4.00 ± 2.31 | 8.86 ± 4.52 | 0 | 0 | 6.00 ± 4.16 | 5.00 ± 2.87 | 5.67 ± 2.03 | 13.92 ± 3.90 |
| PP5 | *Longidorus* | 0 | 0 | 0.67 ± 0.67 | 1.90 ± 1.90 | 2.00 ± 1.15 | 2.12 ± 1.47 | 0 | 0 |

Note: Ba 1, Ba2 and Ba3 represented bacterivore with c-p value of 1, 2 and 3, respectively. Fu2 represented fungivore with c-p value of 2. OP4 and OP5 represented omnivores and predators with c-p value of 4 and 5, respectively. PP 2 and PP3 represented plant parasite with c-p value of 2 and 3, respectively. CK: control;; LG: large gap; MG: medium gap; SG: small gap.

**Table S_4_.** Abundances (Means ± SEs, *N* = 3, Individuals/100 g dry soil) for trophic groups of soil nematodes in different-sized gaps of spruce plantation in the eastern Tibetan Plateau, China

|  | Ba1 | Ba2 | Ba3 | Fu2 | OP4 | OP5 | PP2 | PP3 |
| --- | --- | --- | --- | --- | --- | --- | --- | --- |
| CK | 0.00±0.00 | 15.00±4.04 ab | 1.33±0.67 | 2.67±0.67 | 10.33±0.88 ab | 0.67±0.67 | 11.33±5.46 | 4.00±2.00 |
| SG | 2.67±1.33 | 8.33±1.67 b | 1.33±0.67 | 2.67±0.67 | 18.00±4.04 a | 0.00±0.00 | 12.00±2.52 | 2.00±2.00 |
| MG | 0.67±0.67 | 44.33±8.84 a | 1.33±0.67 | 1.33±0.67 | 14.33±4.91 a | 2.67±0.67 | 27.00±5.86 | 13.33±4.81 |
| LG | 4.00±2.00 | 8.00±2.31 b | 1.33±0.67 | 3.67±1.67 | 2.67±0.67 b | 0.67±0.67 | 6.00±2.00 | 8.67±5.70 |
| P_GS_ | 0.20 | 0.01 | 1.00 | 0.29 | 0.01 | 0.12 | 0.08 | 0.26 |
| P_B_ | 0.45 | 0.44 | 0.82 | 0.21 | 0.04 | 1.00 | 0.69 | 0.35 |

Note: the different lowercase letters in the same column represented significant differences across gaps based on post-hoc test with Tukey’s Honestly Significant Difference (HSD) test at α=0.05 level. Ba 1, Ba2 and Ba3 represented bacterivores with c-p value of 1, 2 and 3, respectively. Fu2 represented fungivores with c-p value of 2. OP4 and OP5 represented omnivores and predators with c-p value of 4 and 5, respectively. PP 2 and PP3 represented plant parasites with c-p value of 2 and 3, respectively. CK: control;; LG: large gap; MG: medium gap; SG: small gap. P_GS_ and P_B_ are *P*-values for linear mixed models with gap size as fixed factor and block as random factor.

**Table S_5_**. Relative abundances (Means± SEs, *N* = 3, %) of functional gulids in distinct-sized gaps of spruce plantation in the eastern Tibetan Plateau, China

|  | Ba1 | Ba2 | Ba3 | Fu2 | OP4 | OP5 | PP2 | PP3 |
| --- | --- | --- | --- | --- | --- | --- | --- | --- |
| CK | 0.00 ± 0.00 | 33.91 ± 8.51 | 2.62 ± 1.32 | 5.82 ± 0.94 a | 23.46 ± 3.14 ab | 1.23 ± 1.23 | 24.20 ± 9.36 | 8.75 ± 4.01 |
| SG | 5.64 ± 2.89 | 17.81 ± 3.68 | 3.07 ± 1.54 | 5.54 ± 0.94 a | 37.58 ± 5.64 a | 0.00 ± 0.00 | 25.91 ± 5.99 | 4.44 ± 4.44 |
| MG | 0.82 ± 0.82 | 41.96 ± 1.64 | 1.19 ± 0.63 | 1.19 ± 0.63 b | 12.91 ± 2.13 b | 2.84 ± 1.07 | 26.34 ± 6.19 | 12.75 ± 4.52 |
| LG | 11.51 ± 5.69 | 23.81 ± 8.46 | 3.89 ± 2.00 | 10.56 ± 4.75 a | 8.02 ± 2.67 b | 1.90 ± 1.90 | 17.30 ± 5.72 | 23.02 ± 13.67 |
| P_GS_ | 0.07 | 0.20 | 0.92 | 0.05 | 0.002 | 0.38 | 0.82 | 0.18 |
| P_B_ | 0.52 | 0.93 | 0.92 | 0.30 | 0.13 | 0.86 | 0.99 | 0.14 |

Note: the different lowercase letters in the same column represented significant differences across gaps based on post-hoc test with Tukey’s Honestly Significant Difference (HSD) test at α=0.05 level. Ba 1, Ba2 and Ba3 represented bacterivores with c-p value of 1, 2 and 3, respectively. Fu2 represented fungivores with c-p value of 2. OP4 and OP5 represented omnivores and predators with c-p value of 4 and 5, respectively. PP 2and PP3 represented plant parasites with c-p value of 2 and3, respectively. CK: control;; LG: large gap; MG: medium gap; SG: small gap. P_GS_ and P_B_ are *P*-values for linear mixed models with gap size as fixed factor and block as random factor.
